# Plant translational reprogramming for stress resilience

**DOI:** 10.3389/fpls.2023.1151587

**Published:** 2023-02-24

**Authors:** Seungmin Son, Sang Ryeol Park

**Affiliations:** National Institute of Agricultural Sciences, Rural Development Administration, Jeonju, Republic of Korea

**Keywords:** abiotic stress, biotic stress, climate change, crop improvement, upstream open reading frame, translational reprogramming

## Abstract

Organisms regulate gene expression to produce essential proteins for numerous biological processes, from growth and development to stress responses. Transcription and translation are the major processes of gene expression. Plants evolved various transcription factors and transcriptome reprogramming mechanisms to dramatically modulate transcription in response to environmental cues. However, even the genome-wide modulation of a gene’s transcripts will not have a meaningful effect if the transcripts are not properly biosynthesized into proteins. Therefore, protein translation must also be carefully controlled. Biotic and abiotic stresses threaten global crop production, and these stresses are seriously deteriorating due to climate change. Several studies have demonstrated improved plant resistance to various stresses through modulation of protein translation regulation, which requires a deep understanding of translational control in response to environmental stresses. Here, we highlight the translation mechanisms modulated by biotic, hypoxia, heat, and drought stresses, which are becoming more serious due to climate change. This review provides a strategy to improve stress tolerance in crops by modulating translational regulation.

## Introduction

Genetic information is transmitted from DNA to proteins through messenger RNA (mRNA) (with certain exceptions—i.e., reverse transcription and prions) according to the central dogma first reported by Francis Crick (Koonin, 2012; Cobb, 2017). Therefore, the strict control of gene expression determines most biological processes, including growth, development, and stress responses. Gene expression occurs in stages, including transcription and translation, that are physically and functionally connected (Orphanides and Reinberg, 2002; Buccitelli and Selbach, 2020). Transcription and translation generally occur simultaneously in prokaryotes lacking membrane-bound organelles, but are spatially separated in eukaryotes. In eukaryotic cells, mRNA is transcribed in the nucleus and then moves to the cytoplasm where translation occurs. The translation mechanism is highly conserved and comprises three major stages: initiation, elongation, and termination (Kapp and Lorsch, 2004). In eukaryotes, translation initiation relies on cap-dependent and cap-independent pathways (Merrick, 2004; Shatsky et al., 2018). Translation is initiated by the assembly of the initiation complex, which consists of eukaryotic initiation factors (eIFs), ribosomes, and the initiator methionyl-transfer RNA (Met-tRNA_i_^Met^) on mRNA (Merrick and Pavitt, 2018). The initiation complex recognizes the initiation codon AUG *via* the anticodon of Met-tRNA_i_^Met^ in the ribosomal peptidyl (P)-site. For translation elongation, amino acids are sequentially and continuously added at the P-site with the help of eukaryotic elongation factors (eEFs) from aminoacyl-tRNAs binding to the aminoacyl (A)-site of the ribosome, and translation terminates when a stop codon (UAG, UGA, or UAA) is located in the A-site and recognized by eukaryotic release factors (Dever et al., 2018).

Climate change is raising global temperatures and shifting regional climates toward greater extremes, including increased aridity in some areas and heavier rainfall in others. In addition, the environmental stresses exacerbated by climate change can impede plant immunity and provide favorable conditions for pathogens (Velasquez et al., 2018). Crop yield and food security are threatened by these increased stresses (Chaudhry and Sidhu, 2022; Son and Park, 2022b). Because plants cannot move to avoid stresses, adaptive changes resulting from alteration of gene expression play critical roles in their survival under extreme stress conditions, and the regulation of gene expression is more crucial to the survival of individuals in plants than in animals. Indeed, not only the numbers of transcription factors but also their rates of expansion are higher in plants (Riechmann et al., 2000; Shiu et al., 2005). Plant protein translation mechanisms are also modulated in response to various stresses (Spriggs et al., 2010; Echevarría-Zomeño et al., 2013; Zlotorynski, 2022). Therefore, investigating the translation mechanisms involved in stress tolerance and engineering them in important crops are essential for sustainable agriculture. Here, we summarize and discuss the translational mechanisms that are modulated by biotic and abiotic stresses.

## Translation reprogramming in response to stress stimulus

The different steps in the translation process are coordinated to ensure survival and efficient use of cellular resources under stress conditions. Stimulus-mediated translational control can be classified into global and gene-specific processes, and its mechanisms are mainly associated with translation initiation and polysome association (Gebauer and Hentze, 2004; Sonenberg and Hinnebusch, 2009). In eukaryotes, stress-induced translational regulation generally results from inhibition of canonical protein biosynthesis driven by cap-dependent translation and induction of stress-associated protein biosynthesis driven by cap-independent translation (Holcik and Sonenberg, 2005; Liu and Qian, 2014). Plant translation processes such as initiation, elongation, and termination are highly conserved, and many components involved in translation mechanisms have been well described in previous reviews (Muench et al., 2012; Roy and Von Arnim, 2013; Browning and Bailey-Serres, 2015; Merchante et al., 2017). Therefore, this review focuses on translation regulation in response to biotic and abiotic stresses.

**Figure 1:**
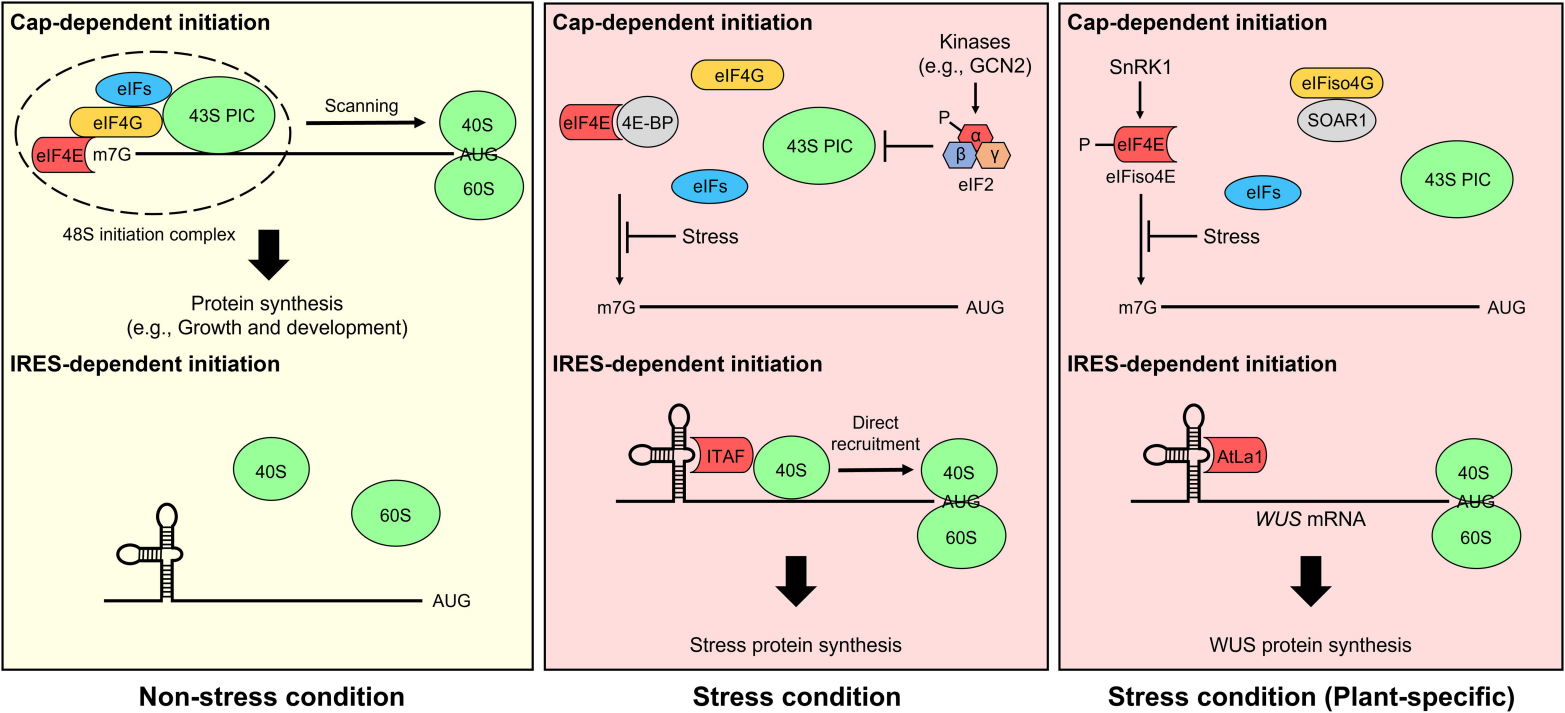
Cap-dependent and internal ribosomal entry site (IRES)-dependent translation initiation mechanisms. Under non-stress conditions, most mRNAs are translated through the cap-dependent translation pathway (top) in eukaryotes, including plants. The eukaryotic initiation factor 4F (eIF4F) complex, including the cap-binding protein eIF4E and the scaffolding protein eIF4G, is assembled on the 5′ cap of mRNA and recruits the 43S pre-initiation complex (PIC) to form the 48S PIC. The complex scans mRNA for an initiation codon. Finally, 60S ribosomes are recruited to form an 80S ribosome initiation complex, and mRNA translation is carried out in earnest. However, cap-dependent translation initiation is prevented under stress conditions. For example, eIF4E-binding protein (4E-BP) and phosphorylation of eIF2α inhibit the formation of eIF4F and the ternary complex, respectively, thereby suppressing cap-dependent translation (Richter and Sonenberg, 2005; Holcik, 2015). In plants lacking 4E-BP, cap-dependent translation is impaired by not only SNF1-related protein kinase 1 (SnRK1)-induced eIF4E and eIF isoform 4E (eIFiso4E) phosphorylation but also the interaction between Suppressor of the ABAR overexpressor 1 (SOAR1) and eIFiso4G (Bi et al., 2019; Bruns et al., 2019). Alternatively, the IRES-dependent translation pathway (bottom) is activated under stress conditions. In general, IRES *trans*-acting factors (ITAFs) mediate IRES-dependent translation in eukaryotes (Komar and Hatzoglou, 2011; Godet et al., 2019). However, this cellular mRNA translation mechanism is largely elusive in plants. Two mRNAs, maize *Heat shock protein 101* (*Hsp101*) and Arabidopsis *WUSCHEL* (*WUS*), are reported to be translated by IRES-dependent translation under stress conditions (Dinkova et al., 2005; Cui et al., 2015b), and Cui et al. only identified Arabidopsis LA protein 1 (AtLa1) binding to the 5′ untranslated region of cellular mRNA as an IRES-dependent translation regulator.

mRNA translation in eukaryotes is initiated through two different mechanisms, cap-dependent ribosome scanning and cap-independent internal ribosome entry, which are regulated by a stress stimulus (**Figure 1**). In non-stress conditions, most mRNAs are translated through the cap-dependent translation pathway. Transcribed nascent pre-mRNA is modified *via* 5′-m7GpppN capping, RNA splicing, and 3′ poly(A) addition, and structural mRNA features are important for their translation (Von Arnim et al., 2014; Sablok et al., 2017). In cap-dependent translation, the cap-binding complex eIF4F consisting of the cap-binding protein eIF4E, the scaffolding protein eIF4G, and the ATP-dependent RNA helicase eIF4A is assembled on the 5′-cap of mRNA, while the poly(A)-binding protein (PABP) interacts with the 3′-poly(A) tail of mRNA (Browning and Bailey-Serres, 2015). In plant, there is also an eIF isoform 4F (eIFiso4F) which consists of eIFiso4E, eIFiso4G, and eIF4A. Subsequently, eIF4F interacts with eIF4B, the eIF4A cofactor, and PABP *via* eIF4G, resulting in mRNA unwinding and circularization. The 43S pre-initiation complex (PIC), which is composed of the 40S ribosome subunit, eIFs (i.e., eIF1, eIF1A, eIF3, and eIF5), and the ternary complex eIF2 (consisting of α, β, and γ subunits)-GTP-Met-tRNA_i_^Met^, interacts with the eIF4F complex and forms the 48S PIC, which scans mRNA in the 5′-to-3′ direction to find an initiation codon. Upon detection of an initiation codon, eIFs, including eIF1 (which is necessary for the fidelity of initiation codon selection), and hydrolyzed eIF2-GDP are released, and eIF5B-GTP and the 60S ribosome subunit are recruited to form the translation-competent 80S ribosome (Browning and Bailey-Serres, 2015; Merchante et al., 2017).

Cap-dependent translation initiation is impaired under stress conditions. For example, in mammals, eIF4E-binding protein (4E-BP) interacts with eIF4E and prevents eIF4F formation by interfering with the binding of eIF4E and eIF4G (Richter and Sonenberg, 2005). Phosphorylation of eIF2α by multiple protein kinases (e.g., General control nondepressible 2 [GCN2]) is another mechanism that inhibits cap-dependent translation in mammals (Holcik, 2015; Hinnebusch et al., 2016). In response to a wide range of signals, stress-activated kinases phosphorylate the Ser51 of eIF2α, which inhibits ternary complex recycling by preventing the guanine nucleotide exchange factor eIF2B-mediated catalysis of GDP to GTP (Buchan and Parker, 2009). However, in plant, an orthologous genes of 4E-BP are absent (Hernandez et al., 2010). Although some proteins (e.g., Lipoxygenase 2, Basic transcription factor 3, Essential for potexvirus accumulation 1, and Conserved binding of eIF4E 1) have been identified as eIF4E- and/or eIFiso4E-binding proteins, their association with cap-dependent translation is unclear (Freire et al., 2000; Freire, 2005; Lázaro-Mixteco and Dinkova, 2012; Wu et al., 2017; Patrick et al., 2018). Even the plant eIF4E-interacting protein CERES has a positive effect on global translation, not a negative effect (Toribio et al., 2019). GCN2-eIF2α module is also controversial in plants (Wang et al., 2022). Alternatively, SNF1-related protein kinase 1 (SnRK1)-mediated eIF4E and eIFiso4E phosphorylation and the interaction of Suppressor of the ABAR overexpressor 1 (SOAR1), involved in abscisic acid signaling, with eIFiso4G inhibit cap-dependent translation initiation in plants (Bi et al., 2019; Bruns et al., 2019).

Although canonical cap-dependent translation is impeded by stress stimuli, selective mRNA translation is induced *via* noncanonical cap-dependent translation and cap-independent translation. eIF3d-mediated noncanonical cap-dependent translation was discovered in human cells (Lee et al., 2015; Lee et al., 2016). eIF3d, a subunit of eIF3, has cap-binding activity and induces the translation of specific mRNAs containing a stem-loop structure that inhibits the recruitment of eIF4F complex in their 5′ untranslated region (5′ UTR). Lamper et al. revealed the translation mechanism regulated by eIF3d for metabolic stress adaptation (Lamper et al., 2020). Under non-stress conditions, Casein kinase 2 (CK2) phosphorylates and inactivates eIF3d. Metabolic stresses, including glucose starvation, inhibit CK2-mediated phosphorylation of eIF3d, resulting in selective mRNA translation through cap-binding of eIF3d. In plant, Toribio et al. showed that CERES interacts with eIF4E, eIFiso4E, eIF4A, eIF3, and PABP and forms noncanonical translation initiation complex in which eIF4G or eIFiso4G is replaced by CERES (Toribio et al., 2019). They suggested the noncanonical complex supports translation initiation and regulates general translation positively when the energy and carbon supply are high. CERES is also involved in a defensive response to turnip mosaic virus regardless of its interaction with eIF4E and eIF4isoE (Toribio et al., 2021). However, the detailed mechanism of selective translation of capped mRNAs by noncanonical cap-dependent translation is unknown in plants.

Internal ribosomal entry site (IRES)-mediated cap-independent translation initiation was first discovered in viral RNA translation of picornavirus (Jackson et al., 1990) and is now recognized as an alternative translation mechanism that commonly occurs in eukaryotic cells under stress conditions (Spriggs et al., 2010; Yang and Wang, 2019). IRESs are frequently identified in cellular mRNAs of genes involved in stress responses and are subdivided into two types: Type I cellular IRESs harbor *cis*-regulatory elements that interact with IRES *trans*-acting factors (ITAFs) for ribosome recruitment (Komar and Hatzoglou, 2011; Godet et al., 2019), while Type II cellular IRESs have short *cis* elements that pair with 18S ribosomal RNA, a component of the 40S ribosomal subunit (Dresios et al., 2006). Since most cellular IRESs are Type I, ITAFs play a critical role in IRES-mediated translation. The mechanisms of IRES-dependent initiation are fairly well studied in animals but have been largely elusive in plants. Only a few studies have revealed the possibility of IRES-dependent translation in plants. Some plant viruses utilize IRES-dependent translation mechanisms for biosynthesis of proteins from their viral RNAs (Jaag et al., 2003; Dorokhov et al., 2006; Karetnikov and Lehto, 2007). In maize (*Zea mays*), the 5′ UTR of *Heat shock protein 101* (*HSP101*) mRNA contains an IRES-like element, and its translation is increased *via* cap-independent translation during heat stress (Dinkova et al., 2005). In Arabidopsis (*Arabidopsis thaliana*), the conserved RNA-binding factor La protein 1 (La1) binds to the 5′ UTR of *WUSCHEL* (*WUS*) mRNA and improves its translation through IRES-dependent initiation under environmental hazard conditions (Cui et al., 2015b). However, further studies are needed to understand the mechanisms of IRES-dependent translation in plants.

Though translation control mechanisms in plants have been extensively studied, how this is regulated by various stresses is largely unknown. Here, we review the current knowledge of the translation mechanisms controlled by biotic, hypoxia, heat, and drought stresses in plants.

## Translational control for pattern-triggered immunity

Severe crop losses caused by various pathogens (e.g., bacteria, fungi, and viruses) threaten global food and nutrition security. Therefore, it is necessary to increase plant innate immunity through effective approach such as translational regulation. Viruses use various strategies to regulate host translation mechanisms for protein synthesis (Jaafar and Kieft, 2019; Geng et al., 2021). Increasing plant antiviral immunity through manipulation of plant translation factors has been well described in previous reviews (Sanfacon, 2015; Hashimoto et al., 2016; Calil and Fontes, 2017; Zhao et al., 2020; Leonetti et al., 2021; Robertson et al., 2022). Therefore, herein, we focus on recent studies that demonstrate notable translation mechanisms associated with non-viral pathogens.

Plant immunity can be classified into pathogen-associated molecular pattern (PAMP)-triggered immunity (PTI), also called pattern-triggered immunity, and effector-triggered immunity (ETI) depending on how pathogens are recognized by the plant (Tsuda and Katagiri, 2010; Faulkner and Robatzek, 2012; Naveed et al., 2020). Sensing of conserved pathogen signatures PAMP, also referred as microbe-associated molecular pattern, by pattern recognition receptors (PRRs) in the plant plasma membrane activates downstream signaling (e.g., Receptor-like cytoplasmic kinases [RLCKs], mitogen-activated protein kinases [MAPKs or MPKs], calcium ion, reactive oxygen species, phytohormones, and transcription factors), and induces PTI which is plant defense response at basal level (Bigeard et al., 2015; DeFalco and Zipfel, 2021). Well-known PRRs are the leucine-rich repeat receptor kinases such as Flagellin sensing 2 (FLS2) and the bacterial translation Elongation factor thermo unstable (EF-Tu) receptor (EFR). Sensing of the 22-amino-acid peptide derived from bacterial flagella, flg22, by FLS2 or the 18- and 26-amino-acid peptides derived from EF-Tu, elf18 and elf26, respectively, by EFR induces elicitor-dependent complex formation through binding with somatic embryogenesis receptor-like kinases (SERKs) such as Brassinosteroid insensitive-associated kinase 1 (BAK1)/SERK3 and BAK1-like 1 (BKK1)/SERK4 (Heese et al., 2007; Schulze et al., 2010; Roux et al., 2011; Sun et al., 2013). The elicitor-dependent complex activates downstream signaling by phosphorylating the RLCK Botrytis-induced kinase 1 (BIK1) and MAPKs, resulting in PTI-related resistance responses (Wang et al., 2020).

**Figure 2:**
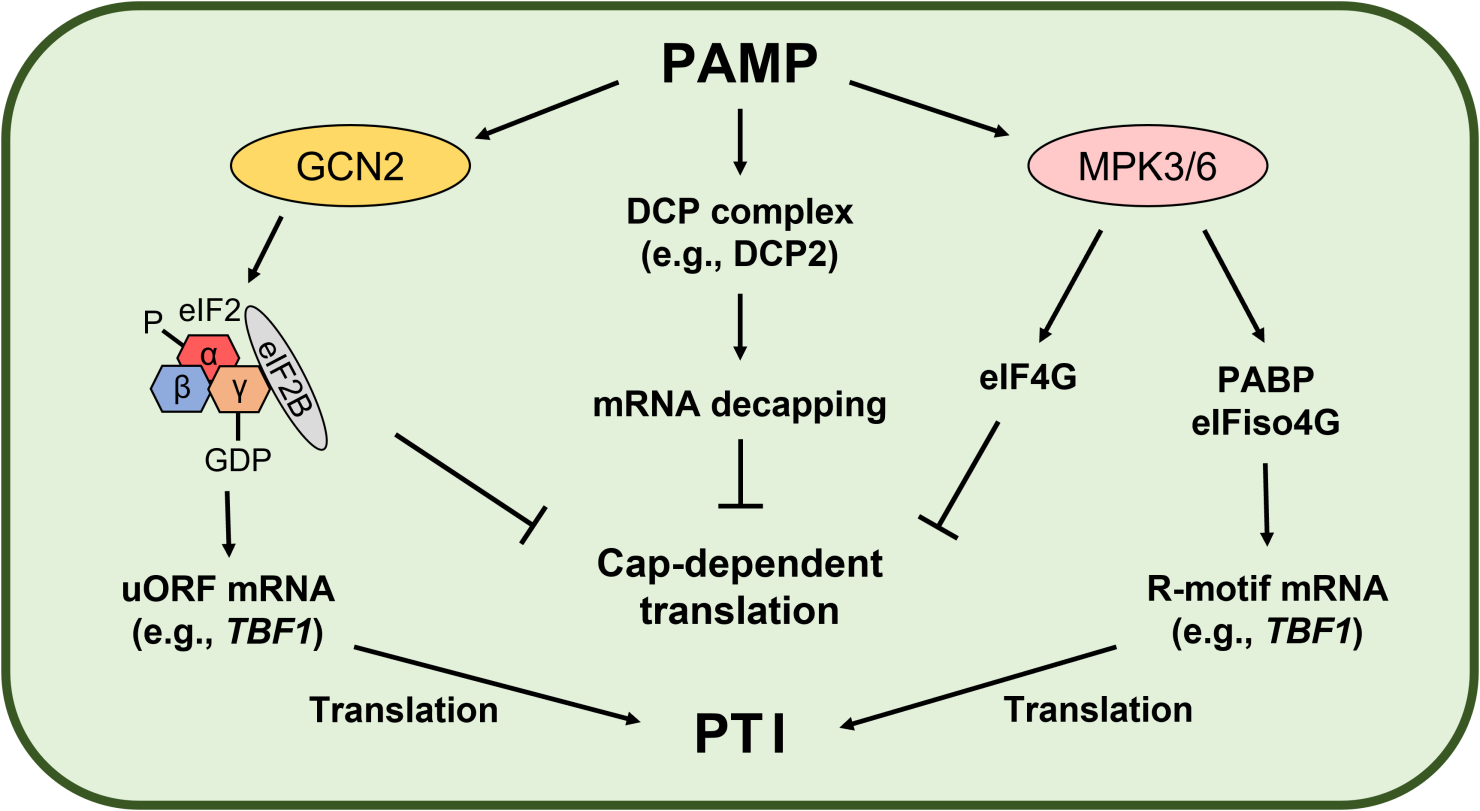
Pathogen-associated molecular pattern (PAMP)-induced translational reprogramming. PAMP-activated General control nondepressible 2 (GCN2) phosphorylates eukaryotic initiation factor 2α (eIF2α), leading to a stable eIF2-GDP-eIF2B complex in Arabidopsis. As a result, formation of the eIF2-GTP-Met-tRNA_i_^Met^ ternary complex required for cap-dependent initiation is inhibited, while translation of defense-related mRNAs with upstream open reading frames (uORFs) conferring negative effects on translation is induced (Pajerowska-Mukhtar et al., 2012; Liu et al., 2019). Wang et al. demonstrated PAMP-mediated translational regulation mechanisms in Arabidopsis. General translation is arrested by PAMP-induced mRNA decapping and Mitogen-activated protein kinase 3 (MPK3)- and MPK6-mediated eIF4G phosphorylation (Wang et al., 2022). On the other hand, MPK3- and MPK6-mediated phosphorylation of the poly(A)-binding protein (PABP) and eIF isoform 4G (eIFiso4G) promotes PABP binding with the R-motif and eIFiso4G to drive PABP-eIFiso4G-mediated translation (Wang et al., 2022). As a result, general mRNA translation is repressed but translation of selective defense-related mRNAs is induced to promote the plant immune response (PAMP-triggered immunity, PTI).

GCN2 is a conserved serine/threonine protein kinase that participates in stress signal transduction, including nutrient starvation and immune responses (Tsalikis et al., 2013; Lokdarshi and von Arnim, 2022). Since eIF2α phosphorylation results in a stable phosphor (P)-eIF2-GDP-eIF2B complex, GCN2-mediated eIF2α phosphorylation arrests global cellular mRNA translation but induces selective translation of stress-responsive mRNAs (e.g., *GCN4* in yeast and *Activating transcription factor 4* in mammals) harboring upstream open reading frames (uORFs) (Dever, 2002). In plants, GCN2 regulates biotic and abiotic stress responses and also stress-induced eIF2α phosphorylation (Liu et al., 2015; Terry et al., 2015; Lokdarshi and von Arnim, 2022). Izquierdo et al. suggested that GCN1 is important for translation regulation and innate immunity in plants, while GCN2 and eIF2α phosphorylation are not (Izquierdo et al., 2018). However, Liu et al. showed that the bacteria-activated GCN2-eIF2α module induces translation of the heat-shock factor-like transcription factor *TL1-binding factor 1* (*TBF1*) mRNA and plant immunity (Liu et al., 2019). TBF1 is a key regulator of the plant growth-defense tradeoff. TBF1 binds to the *TL1 cis* element (GAAGAAGAA) and controls salicylic acid- and elf18-induced transcriptional reprogramming (Pajerowska-Mukhtar et al., 2012). Interestingly, *TBF1* mRNA has two uORFs conferring negative effects on its translation. Pathogen challenge results in eIF2α phosphorylation and derepresses *TBF1* mRNA translation (Pajerowska-Mukhtar et al., 2012). Therefore, the translationally regulated GCN2-eIF2α-TBF1 modules may play a key role in PTI (**Figure 2**).

Global translatome profiling using ribosome footprinting revealed that PTI triggers translational reprogramming to enhance plant immunity (Xu et al., 2017a). elf18 treatment increased the translational efficiency of 448 genes but decreased that of 389 genes in Arabidopsis. Interestingly, the R-motif, which consists almost exclusively of purines, is enriched on the 5′ UTR of the mRNAs, including *TBF1* mRNA, whose translational efficiency is upregulated by elf18. MPK3, MPK6, and PABP is important for R-motif-mediated selective translation during PTI (Xu et al., 2017a). Processing bodies (P-bodies) also play an important role in PTI. P-bodies are cytoplasmic granules consisting of messenger ribonucleoprotein and are involved in translation arrest and mRNA decay (Decker and Parker, 2012; Maldonado-Bonilla, 2014). In eukaryotes, most mRNAs are degraded *via* the deadenylation-dependent mRNA decay pathway (Garneau et al., 2007). After deadenylation, mRNA is degraded immediately by 3′–5′ exonucleases in exosomes or decapped for later degradation by 5′–3′ exonucleases in P-bodies. In Arabidopsis, the decapping complex—involving mRNA-decapping enzyme Decapping protein 2 (DCP2), the co-activator DCP1, and the scaffold protein Varicose—is localized in P-bodies (Xu et al., 2006). However, PAMP-activated MPK3 and MPK6 phosphorylate DCP1, resulting in disassociation of DCP1 and DCP2 and rapid P-body disassembly within 15 to 30 minutes after flg22 treatment (Yu et al., 2019). Yu et al. suggested that phosphorylated DCP1 leads to mRNA degradation of negative regulators of plant immunity by 5′-to-3′ Exoribonuclease 4 (XRN4), an ortholog of yeast XRN1, and translation of defense-related mRNAs stored in P-body assemblies to promote the plant immune response.

Recently, the PTI-induced translational reprogramming mechanism was demonstrated in Arabidopsis (**Figure 2**). As global translation repression mechanism, elf18 induces DCP-complex-mediated mRNA decapping and MPK3- and MPK6-mediated eIF4G phosphorylation (Wang et al., 2022). These mechanisms compromise translation of mRNA related to growth and defense. However, for selective translation control during PTI, defense-related mRNAs containing the R-motif are translated through a PABP-eIFiso4G-mediated cap-independent pathway (Wang et al., 2022). During PTI, PAMP-activated MPK3 and MPK6 phosphorylate PABP and eIFiso4G using Receptor for activated C kinase 1 (RACK1) as a scaffold, which enhances PABP binding with the R-motif in mRNAs and with eIFiso4G, resulting in translation of defense-related mRNAs for innate immunity.

## Translational control for effector-triggered immunity

Plant nucleotide-binding and leucine-rich repeat (NLR) proteins are classified into three groups (i.e., Toll/interleukin-1 receptor-like NLR, coiled coil NLR [CNL], and Resistance to powdery mildew 8-like NLR) based on their N-terminal domain and play various roles as sensors, helpers, and executors in ETI signaling (Son et al., 2021). Direct or indirect recognition of specific effectors (pathogen-secreted proteins that repress PTI and promote infection) by NLRs activates downstream signaling and triggers prolonged and robust ETI resistance responses (Cui et al., 2015a; Lolle et al., 2020).

Two CNLs, Resistance to *Pseudomonas syringae* pv. *maculicola* 1 (RPM1) and Resistance to *P.syringae* 2 (RPS2), are activated by post-translational modification of the plasma-membrane-localized RPM1-interacting protein (RIN4), which interacts with CNLs as well as *P. syringae* avirulence (Avr) effectors (e.g., AvrB, AvrRpm1, and AvrRpt2). In an incompatible interaction, AvrB and AvrRpm1 interact with RIN4, which is inactivated by the prolyl-peptidyl isomerase Rotamase cyclophilin 1, leading to RPM1-induced protein kinase-mediated RIN4 phosphorylation and RPM1 activation for ETI (Mackey et al., 2002; Liu et al., 2011; Li et al., 2014). On the other hand, the interaction of AvrRpt2 with RIN4 leads to RIN4 cleavage into AvrRpt2 cleavage products and activates RPS2 for ETI (Axtell and Staskawicz, 2003; Mackey et al., 2003; Takemoto and Jones, 2005).

Global translatome analysis revealed that RPM1- and RPS2-induced ETI are involved in translation reprogramming. Meteignier et al. showed that AvrRpm1-mediated RPM1 activation modulates the translational status of hundreds of mRNAs involved in growth-defense tradeoffs (Meteignier et al., 2017). RPM1 increases translation efficiency of defense-related mRNAs (e.g., *BIG*, *Phosphorylcholine cytidylyltransferase 2* and *Redox responsive transcription factor 1*/*Ethylene response factor 109*) but decreases translation efficiency of mRNAs involved in growth and/or functioning as negative regulators of defense (e.g., *Target of rapamycin* [*TOR*], *CBL-interacting protein kinase 5*, and *Homolog of BEE2 interacting with IBH 1*). The TOR kinase is the conserved master regulator of the energy signaling pathway regulating growth and metabolism (Wullschleger et al., 2006). In mammals, TOR-mediated phosphorylation of 4E-BP1, Ribosomal protein S6 kinase (S6K), and La-related protein 1 has critical roles in mRNA translation (Yang et al., 2022). Although 4E-BPs remain elusive in plants, the TOR-S6K-ribosomal protein S6 (rpS6) pathway is conserved (Obomighie et al., 2021). Unlike most eukaryotes, which harbor two distinct TOR complexes, plants have only one TOR complex (TORC), consisting of TOR, Regulatory-associated protein of TOR (RAPTOR), and Lethal with sec thirteen protein 8 (LST8) (Maegawa et al., 2015). In Arabidopsis, light and auxin activate TOR-S6K-rpS6 modules to enhance translation (Schepetilnikov et al., 2013; Chen et al., 2018). In response to auxin, eIF3h phosphorylation by TOR-activated S6K1 enhances translation reinitiation of mRNAs with uORFs (Schepetilnikov et al., 2013). Furthermore, the TOR-S6K module phosphorylates MA3-domain-containing translation regulatory factor 1 (MRF1), which then interacts with eIF4A to enhance MRF1 ribosome association for mRNA translation (Lee et al., 2017). Therefore, RPM1-mediated TOR repression may be an important translational mechanism for the growth-defense tradeoff (Meteignier et al., 2017). AvrRpt2-mediated RPS2 activation also triggers translational regulation for ETI. Translational regulation by RPS2 differs from PTI-induced translational reprogramming (Yoo et al., 2020). RPS2-mediated ETI induces TBF1 translation later than PTI does, and is involved in targeted changes in active translation of specific mRNAs instead of general translation inhibition. In addition, unlike with PTI-induced translational regulation, there is a strong correlation between transcriptional and translational changes during RPS2-mediated ETI. There is overlap between RPS2-mediated and RPM1-mediated translational responses (Yoo et al., 2020). For example, 80% of upregulated and 75% of downregulated genes from the RPS2-mediated translational response overlapped with those of the RPM1-mediated translational response. Moreover, RPS2- and RPM1-mediated ETI contribute to metabolic dynamics *via* translational regulation (Meteignier et al., 2017; Yoo et al., 2020). Therefore, ETI-mediated translational regulation in metabolic pathways is important for plant immunity. However, the detailed mechanisms by which ETI regulates protein translation have not been elucidated.

## Translation mechanisms regulated by hypoxia in plants

Hypoxia in plants is caused by flooding, submergence, and soil compaction, and its effect on modulating mRNA translation has been well studied. Hypoxia leads to energy deficiency in plant cells by inhibiting mitochondrial respiration, and plants must redistribute their energy reserves by restricting energy-consuming processes and inducing energy-conserving processes (Geigenberger, 2003; Fukao and Bailey-Serres, 2004; Bailey-Serres and Voesenek, 2008). Since mRNA translation uses an enormous amount of energy, it has to be modified under hypoxic conditions (Kafri et al., 2016; Chee et al., 2019).

In response to hypoxia, maize plants repress the translation of aerobic proteins and increase the translation of anaerobic proteins to confer flooding tolerance (Sachs et al., 1980; Sachs et al., 1996). Hypoxia-mediated selective translation induces protein biosynthesis of maize Alcohol dehydrogenase 1, and the 5′ and 3′ UTRs of its mRNA are necessary for its translation (Bailey-Serres and Dawe, 1996). In maize roots, hypoxia induces eIF4A and eIF4E phosphorylation (Webster et al., 1991; Manjunath et al., 1999), and quantitative analysis of ribosomal complexes in maize seedlings showed that translational machineries involved in initiation and elongation (i.e., eIF4E, eIF4A, eIF4B, and eEF2) are significantly more phosphorylated under oxygen deprivation (Szick-Miranda et al., 2003). In Arabidopsis, although the translation of most cellular mRNA is impaired by hypoxia due to reduced ribosome recruitment, polysome association of some stress-induced mRNAs containing a low GC nucleotide content in the 5′ UTR is increased (Branco-Price et al., 2005). Restriction of global translation in response to hypoxia is necessary for energy conservation (Branco-Price et al, 2008), and it is mainly regulated at the translation initiation level (Juntawong et al., 2014). In addition, hypoxia-induced Oligouridylate binding protein 1C binds mRNAs and forms protein-RNA complexes named stress granules, resulting in global translational arrest *via* mRNA sequestration (Lee et al., 2011; Sorenson and Bailey-Serres, 2014).

**Figure 3:**
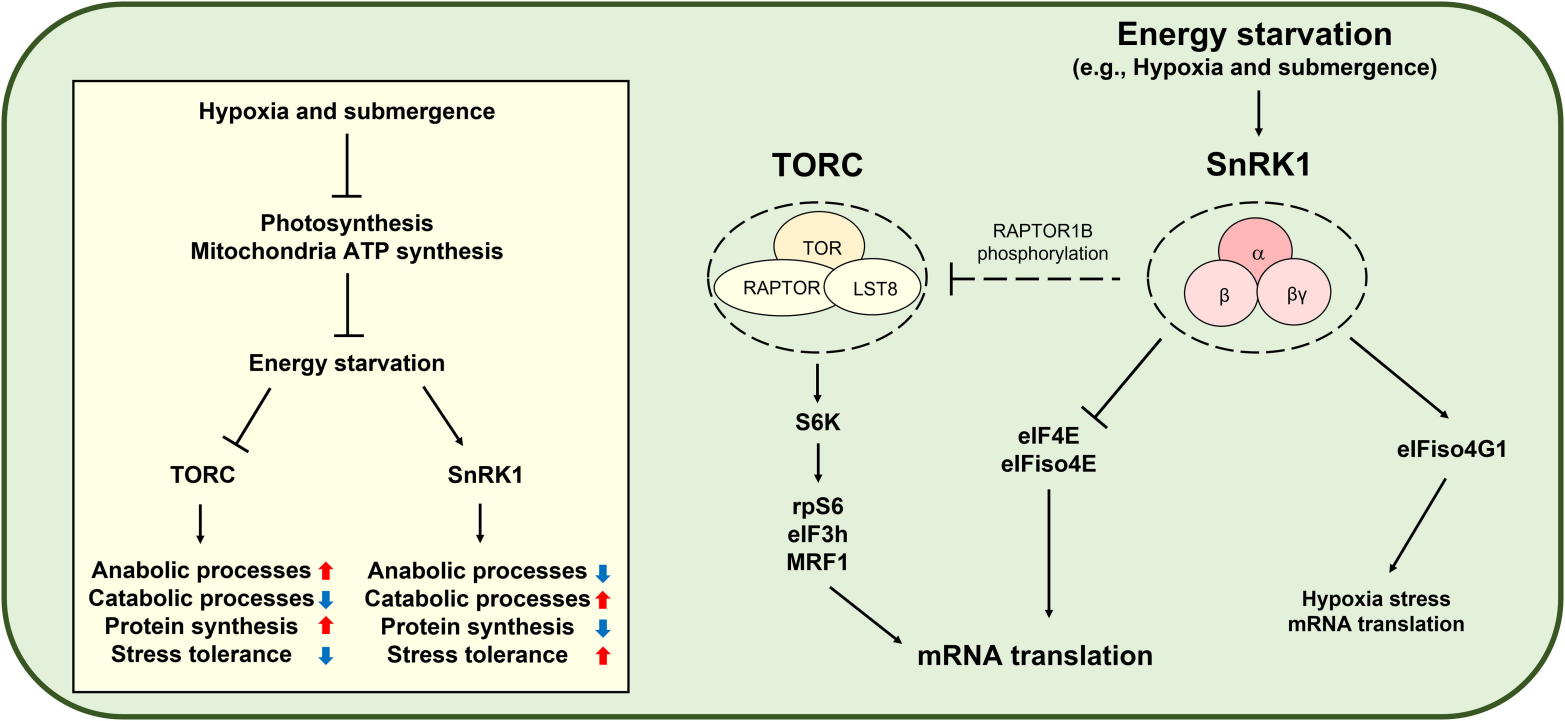
SNF1-related protein kinase 1 (SnRK1)-mediated translational control under hypoxia. Hypoxia and submergence in plants induce energy starvation and activate SnRK1, which consists of an α catalytic subunit and two regulatory subunits, β and βγ. SnRK1 reduces anabolic processes, including protein biosynthesis, while inducing catabolic processes and stress tolerance. Since mRNA translation is an energy-consuming process, SnRK1 modulates mRNA translation. Under submergence conditions, SnRK1 phosphorylates eukaryotic initiation factor 4E (eIF4E) and eIF isoform 4E (eIFiso4E) to repress global mRNA translation (Bruns et al., 2019), whereas SnRK1-mediated eIFiso4G1 phosphorylation induces translation of mRNAs of hypoxia-response genes (Cho et al., 2019). The Target of rapamycin complex (TORC) consists of Target of rapamycin (TOR), Regulatory-associated protein of TOR (RAPTOR), and Lethal with sec thirteen protein 8 (LST8) and is a conserved master regulator of energy signaling, including protein biosynthesis. TOR-activated ribosomal protein S6 kinase (S6K) phosphorylates ribosomal protein S6 (rpS6), eukaryotic initiation factor 3h (eIF3h), and MA3-domain-containing translation regulatory factor 1 (MRF1) to promote mRNA translation (Schepetilnikov et al., 2013; Lee et al., 2017; Chen et al., 2018). The SnRK1 ortholog in mammals is AMP-activated protein kinase (AMPK). AMPK-mediated TORC inhibition is well established in mammals. However, although studies have suggested SnRK1-mediated TOR inhibition through RAPTOR1B phosphorylation (Nietzsche et al., 2016; Nukarinen et al., 2016), it remains obscure in plants.

AMP-activated protein kinase (AMPK) is the evolutionarily conserved energy stress signaling master regulator controlling cellular energetic homeostasis in animals (Trefts and Shaw, 2021). AMPK inhibits mRNA translation in multiple steps by inhibiting TOR, eEF2, and RNA biding proteins (Liu and Chern, 2021). SnRK1 is the plant ortholog of AMPK and controls plant growth, stress tolerance, and metabolism to cope with constantly fluctuating environments (Baena-Gonzalez et al., 2007; Cho et al., 2012; Son et al., 2023). Although there is no clear evidence, some studies suggest that SnRK1-mediated TOR inhibition may occur *via* RAPTOR1B phosphorylation (Nietzsche et al., 2016; Nukarinen et al., 2016). Remarkably, SnRK1-mediated eIF4E and eIFiso4E phosphorylation attenuates general translation (Bruns et al., 2019). Furthermore, SnRK1 activity represses canonical protein biosynthesis without significantly changing transcript levels and protein stability (Son et al., 2022). However, under submergence conditions, SnRK1-mediated eIFiso4G1 phosphorylation confers translational enhancement of specific mRNAs, including those of core hypoxia-response genes (Cho et al., 2019). Therefore, SnRK1 plays a key role in global translational repression and specific mRNA translation under energy deficiency conditions (**Figure 3**). Moreover, ethylene regulates translational dynamics through both a noncanonical ethylene-signaling-activated GCN2-eIF2α module and canonical Ethylene insensitive 2-mediated ethylene signaling during submergence (Cho et al., 2022).

## Translation mechanisms regulated by heat and drought stress in plants

To help plants adapt and survive under elevated temperature, canonical protein translation is significantly suppressed, while the translation of HSPs, which prevent protein denaturation and aggregation typically *via* a chaperone, is induced (Key et al., 1981; Nover et al., 1989; Ndimba et al., 2005; Matsuura et al., 2010; Bita and Gerats, 2013). In carrot (*Daucus carota*), heat stress impedes translation initiation and hampers generation of the 5′ cap and 3′ poly(A) tail conferring mRNA translation (Gallie et al., 1995). eIF4A and eIF4B are phosphorylated during heat stress in wheat (*Triticum aestivum*), but eIF4F, eIFiso4F, eIF2α, eIF2β, and PABP are not (Gallie et al., 1997). In Arabidopsis, mRNA sequence features, such as the GC content of the 5′ UTR and cDNA length, are important elements conferring heat-induced selective mRNA translation (Yangueez et al., 2013). Under elevated temperature, Arabidopsis XRN4 degrades mRNAs encoding HSP70 binding proteins and hydrophobic N-terminal proteins in polysomes, triggering ribosome pausing (Merret et al., 2015). eIF5B contributes to the biosynthesis of stress-protective proteins under high temperature, and eIF5B is important for heat stress tolerance in Arabidopsis (Zhang et al., 2017). Moreover, Bonnot and Nagel showed that the circadian clock and heat stress interact to prioritize the translation of the mRNA pool in Arabidopsis (Bonnot and Nagel, 2021). Translation of heat- or abiotic-stress-related mRNAs, including *HSP90-3*, was significantly upregulated under high temperature. They also suggested that transcription factors, including Cycling DOF factor, MYB-related, and B-box families, play important roles in heat stress-mediated plant growth dynamics dependent on the circadian clock.

Dehydration also changes mRNA translational efficiencies in plants, including Arabidopsis, maize, and soybean (*Glycine max*) (Hsiao, 1970; Dhindsa and Cleland, 1975; Rhodes and Matsuda, 1976; Bensen et al., 1988; Hurkman and Tanaka, 1988; Mason et al., 1988; Valluri et al., 1989; Bray, 1990; Kawaguchi et al., 2003; Kawaguchi et al., 2004). In Arabidopsis, polysome association of 71% of 2,136 genes is significantly decreased under dehydration conditions (Kawaguchi et al., 2004), and mRNA sequence features (i.e., 5′-UTR GC content, initiation codon context, and ORF length) influence the dehydration-mediated differential mRNA translation (Kawaguchi and Bailey-Serres, 2005). Furthermore, translatome profiling revealed that mRNA sequence features as well as uORFs are important for dynamic translational changes under drought conditions in maize (Lei et al., 2015). In rice (*Oryza sativa*), more than 50% of the genes encoding ribosomal proteins (e.g., rpS4, rpS10, rpS18a, rpL6, rpL7, rpL23A, rpL24, and rpL31) are upregulated in response to drought, and overexpressing *rpL23A* confers rice drought tolerance (Moin et al., 2017). In addition, silencing of *rpL14B* decreases drought tolerance in cotton (*Gossypium hirsutum*) (Shiraku et al., 2021). However, although ribosomal proteins are abundant RNA-binding proteins involved in ribosome structure and protein biosynthesis, they also have additional functions (Warner and McIntosh, 2009). Therefore, it is not clear whether the drought effects associated with ribosomal proteins are related to mRNA translation.

## uORF-mediated translational control for crop improvement

The development of stress-resilient crops is needed to cope with the environmental extremes caused by climate change and the increase in food demand due to world population expansion. General protein translation is significantly decreased under stressful conditions, while translation of specific stress-associated proteins is increased (Muench et al., 2012; Merchante et al., 2017). uORFs are a conserved structure found in 30–60% of eukaryotic transcripts, including plant transcripts, and are important for mRNA translation (Von Arnim et al., 2014; Chew et al., 2016). Most mRNAs of stress-related genes upregulated by a specific stress stimulus contain uORF sequences in plants (Ebina et al., 2015; Hayashi et al., 2017). Therefore, uORF-mediated translational control represents a promising method to improve the stress resilience of crops.

Engineering plants to express specific stress-responsive genes can enhanced stress resilience (Kamthan et al., 2016). However, it can also reduce plant growth and yield due to tradeoff effects (Gurr and Rushton, 2005; Huot et al., 2014; Waadt et al., 2022). These fitness costs can be resolved by uORF-mediated translational regulation. For example, Xu et al. developed a TBF1 cassette using the *TBF1* promoter and two uORFs on *TBF1* mRNA that conferred pathogen-inducible translational control to overcome the fitness costs of the plant immune response (Xu et al., 2017b). They showed that *pTBF1:uORFs*-driven expression of Arabidopsis *Nonexpressor of pathogenesis-related genes 1*, encoding a master regulator of salicylic-acid-mediated defense signaling that contributes to broad-spectrum resistance, increased rice resistance to the bacterial blight pathogen *Xanthomonas oryzae* pv. *oryzae* and the rice blast pathogen *X. oryzae* pv. *oryzicola* without fitness penalties. Therefore, developing uORF-based promoters triggering mRNA translation in response to a specific stress stimulus is an important strategy for improving crop stress resistance.

The development of the clustered regularly interspaced short palindromic repeats (CRISPR)/CRISPR-associated nuclease 9 (Cas9) technology was an innovation that allows efficient genome editing without the presence of transgenes or the regulatory issues associated with genetically modified crops (Son and Park, 2022a). Not only coding regions but also promoter regions regulating gene expression are good targets for genome editing for crop improvement (Hua et al., 2019). Since most uORFs inhibit mRNA translation *via* ribosome stalling, ribosome disassociation, uORF translation past the initiation codon, and mRNA decay, mutation of uORFs by CRISPR/Cas9 can change translation efficiency (Young and Wek, 2016; Um et al., 2021). Indeed, crop improvement through CRISPR/Cas9-mediated editing of uORFs regulating mRNA translation has been reported. Lettuce (*Lactuca sativa*) *GDP-L-galactose phosphorylase 2* (*LsGGP2*) uORF editing enhanced ascorbic acid (vitamin C) contents and tolerance to oxidative stress in iceberg lettuce (Zhang et al., 2018). Mutation of *GGP1* uORFs by CRISPR/Cas9 also increased the ascorbic acid content in wild tomato (*Solanum pimpinellifolium*) accession LA1589 and bacterial spot disease resistance (Li et al., 2018). Editing of the *Phosphate 1* uORF induced root inorganic orthophosphate (Pi) accumulation and Pi deficiency tolerance in Arabidopsis (Reis et al., 2020). Strawberry (*Fragaria vesca*) *S1-group basic leucine zipper protein 1.1* editing increased the sugar content (Xing et al., 2020). CRISPR/Cas9-mediated mutation of *Heading date 2* delayed flowering time in rice (Liu et al., 2021). Previously, it was difficult to improve crop traits through uORF editing due to technical limitations, but more convenient methods are continuously being developed (Si et al., 2020). Therefore, uORF-mediated translational control is expected to become accessible to many scientists and contribute to stress resilience in crops.

## Conclusions

Global climate change exacerbates abiotic and biotic stresses in crops, and improving crop stress resilience is essential for sustainable agriculture. Although advances in biotechnology, including CRISPR/Cas9 gene editing systems, have facilitated crop improvement, they require identification and characterization of genes and their regulatory networks. Since translational control is an efficient way to increase resistance to various stresses, it is essential to understand the mechanisms controlling protein biosynthesis. Numerous global translatome profiling technologies, such as polysome profiling, ribosome profiling, translating ribosome affinity purification, and 3′ ribosome-profiling sequencing, have been developed and utilized to study plant translation (Ingolia et al., 2009; Heiman et al., 2014; Mazzoni-Putman and Stepanova, 2018; Zhu et al., 2021) and have facilitated important advances in plant biology. Therefore, we have provided here an overview of the current understanding of protein translational control in response to pathogens, hypoxia, heat, and drought stresses in plants, and discussed a crop improvement strategy based on translational regulation through editing of uORFs. Though the translational control mechanisms involved in stress responses need further study, their exploration and application in crop breeding are important steps toward developing stress tolerant crop cultivars without fitness costs.

## Author contributions

SS conceptualized and wrote the manuscript. SRP supervised. All authors contributed to the article and approved the submitted version.
